# Severe acute myocarditis induced by 5-fluorouracil with successful rechallenge after multidisciplinary cardio-oncology evaluation: a case report

**DOI:** 10.1093/ehjcr/ytag285

**Published:** 2026-04-24

**Authors:** Alessandra Greco, Anna Pagani, Sara Modugno, Martina Iengo, Gianpiero Rizzo, Carlotta Faverio, Paolo Pedrazzoli, Leonardo De Luca

**Affiliations:** Cardiology Unit, Fondazione IRCCS Policlinico San Matteo, Piazzale Golgi 19, 27100 Pavia, Italy; Oncology Unit, Fondazione IRCCS Policlinico San Matteo, Piazzale Golgi 19, 27100 Pavia, Italy; Cardiology Unit, Fondazione IRCCS Policlinico San Matteo, Piazzale Golgi 19, 27100 Pavia, Italy; Cardiology Unit, Fondazione IRCCS Policlinico San Matteo, Piazzale Golgi 19, 27100 Pavia, Italy; Oncology Unit, Fondazione IRCCS Policlinico San Matteo, Piazzale Golgi 19, 27100 Pavia, Italy; Oncology Unit, Fondazione IRCCS Policlinico San Matteo, Piazzale Golgi 19, 27100 Pavia, Italy; Oncology Unit, Fondazione IRCCS Policlinico San Matteo, Piazzale Golgi 19, 27100 Pavia, Italy; Cardiology Unit, Fondazione IRCCS Policlinico San Matteo, Piazzale Golgi 19, 27100 Pavia, Italy

**Keywords:** 5-fluorouracil myocarditis, Rechallange, Cardio-oncology team, Case report

## Abstract

**Background:**

Fluoropyrimidines are among the most common chemotherapeutic agents associated with cardiotoxicity, typically presenting as angina due to coronary vasospasm. Severe manifestations such as myocarditis and cardiogenic shock are rare, and rechallenge after cardiotoxicity is generally discouraged. We report a case of acute 5-fluorouracil–induced myocarditis with successful rechallenge after multidisciplinary evaluation.

**Case summary:**

A 30-year-old man with unresectable rectal adenocarcinoma developed acute cardiotoxicity during the first FOLFOX-6 cycle, presenting with abdominal pain and palpitations. ECG showed atrial fibrillation and ST-segment elevation. Echocardiography revealed severe biventricular dysfunction with elevated cardiac biomarkers. Coronary angiography excluded obstructive disease, and cardiac magnetic resonance confirmed acute myocarditis with diffuse oedema and non-ischaemic late gadolinium enhancement. Guideline-directed heart failure therapy led to rapid functional recovery within days. Following multidisciplinary cardio-oncology assessment, chemotherapy rechallenge under continuous monitoring was performed without recurrence of major adverse events. Subsequent cycles were well tolerated, and follow-up CMR showed preserved ventricular function with residual non-ischaemic LGE.

**Discussion:**

This case highlights the importance of early recognition of fluoropyrimidine cardiotoxicity, the role of multidisciplinary cardio-oncology management, and the potential feasibility of rechallenge in carefully selected patients.

## Graphical Abstract

**Graphical Abstract:**
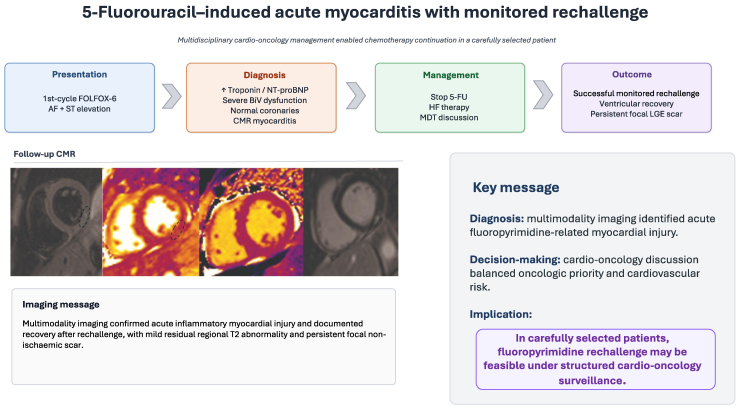
Clinical course, diagnostic work-up, management, and follow-up of 5-fluorouracil-related acute myocarditis with successful monitored rechallenge. During first-cycle FOLFOX-6, the patient developed atrial fibrillation, ST-segment elevation, biomarker rise, and severe reversible biventricular dysfunction. Multimodality cardiovascular assessment supported the diagnosis of acute inflammatory myocardial injury. After 5-fluorouracil discontinuation and heart failure therapy, rapid recovery was observed. Following multidisciplinary cardio-oncology discussion, chemotherapy was safely reintroduced under structured surveillance. Follow-up CMR showed recovery of ventricular function, mild residual regional T2 abnormality, and persistent focal non-ischaemic late gadolinium enhancement consistent with residual post-inflammatory scar.

Learning points

- Fluoropyrimidine cardiotoxicity can present as severe acute myocarditis even in patients without known cardiovascular disease or baseline risk factors.
- Early recognition of 5-fluorouracil cardiotoxicity and prompt cardiologic management may allow complete recovery of ventricular function.
- Structured multidisciplinary cardio-oncology assessment can enable safe chemotherapy rechallenge in selected patients after severe cardiotoxicity.

## Introduction

Fluoropyrimidines, including 5-fluorouracil and capecitabine, are widely used chemotherapeutic agents^1^ but are associated with a spectrum of cardiovascular toxicities ranging from angina and arrhythmias to rare cases of myocarditis and cardiogenic shock.^2,3^ Although coronary vasospasm is considered the most common mechanism, the pathophysiology of fluoropyrimidine cardiotoxicity remains incompletely understood.^4^ Early recognition is essential because prompt discontinuation of therapy and cardiologic management may prevent severe complications and allow treatment continuation in selected patients.^5^

Here, we report a case of severe acute myocarditis occurring during first-cycle FOLFOX-6 chemotherapy in a 30-year-old man with unresectable rectal adenocarcinoma, highlighting the diagnostic value of comprehensive cardiac assessment and the importance of multidisciplinary cardio-oncology management.

Clinical course, diagnostic work-up, management, and follow-up of 5-fluorouracil-related acute myocarditis with successful monitored rechallenge. During first-cycle FOLFOX-6, the patient developed atrial fibrillation, ST-segment elevation, biomarker rise, and severe reversible biventricular dysfunction. Multimodality cardiovascular assessment supported the diagnosis of acute inflammatory myocardial injury. After 5-fluorouracil discontinuation and heart failure therapy, rapid recovery was observed. Following a multidisciplinary cardio-oncology discussion, chemotherapy was safely reintroduced under structured surveillance. Follow-up CMR showed recovery of ventricular function, mild residual regional T2 abnormality, and persistent focal non-ischaemic late gadolinium enhancement consistent with residual post-inflammatory scar.

## Summary figure

**Figure:**
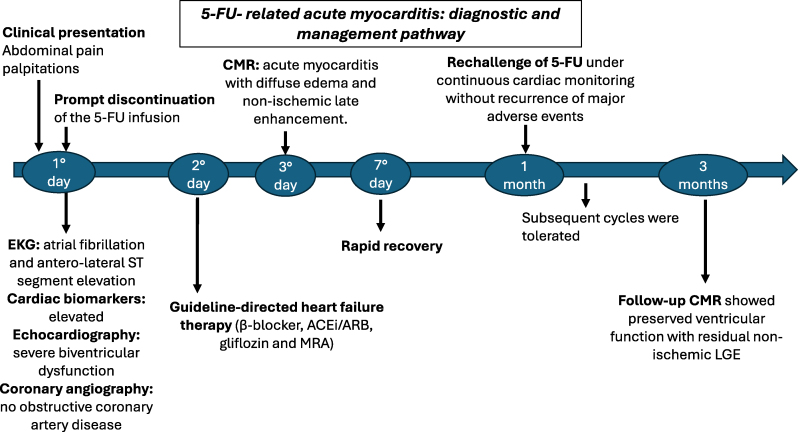


## Case presentation

A 30-year-old man with unresectable rectal adenocarcinoma (cT3d cN2b cM0, pMMR, RAS/BRAF wild type; NGS showing a likely pathogenic TP53 mutation; HER2 negative) was considered eligible for chemotherapy with the FOLFOX-6 regimen (5-fluorouracil, oxaliplatin, and folinic acid). At baseline, he was classified as low cardiovascular risk, with a normal electrocardiogram and unremarkable laboratory tests.

**Figure 1:**
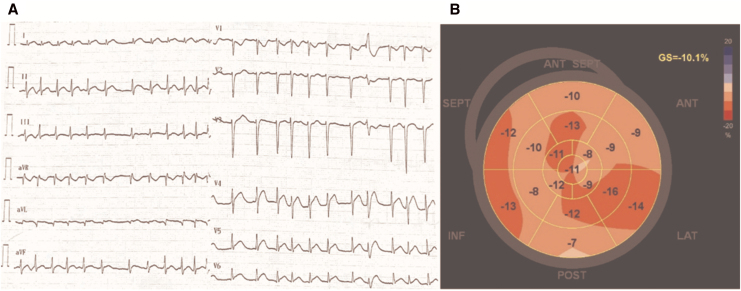
Baseline electrocardiographic and echocardiographic findings. *(A*) ECG showing atrial fibrillation with rapid ventricular response and diffuse ST-segment elevation, more pronounced in the lateral leads. *(B*) Transthoracic echocardiography with global longitudinal strain (GLS) assessment: polar map of left ventricular longitudinal strain demonstrating severely reduced global systolic function with a global longitudinal strain (GLS) of −10.1%. Segmental strain values are diffusely impaired without a regional ischaemic distribution, consistent with severe global myocardial dysfunction. Findings are associated with severe biventricular systolic impairment.

During the final hours of continuous 5-fluorouracil infusion, the patient developed abdominal pain, nausea, and palpitations. Electrocardiography (ECG) showed atrial fibrillation with a ventricular rate of 150 bpm and anterolateral ST-segment elevation (*Figure 1A*), prompting immediate discontinuation of the infusion. Ten minutes after stopping 5-fluorouracil, the patient spontaneously cardioverted to sinus rhythm at 93 bpm, with persistent ST-segment elevation and no reciprocal changes.

Transthoracic echocardiography revealed severe biventricular systolic dysfunction (LVEF 27%, FAC 27%, TAPSE 11 mm, LV-GLS −10%; *Figure 1B*), global hypokinesia, and severe mitral regurgitation. Despite these findings, the patient remained haemodynamically stable, with normal blood pressure, adequate peripheral perfusion, and no detectable lactate on arterial blood gas analysis.

Laboratory testing showed marked elevations of troponin (852 ng/L; reference <47 ng/L), NT-proBNP (3115 ng/L; reference <150 ng/L), and CRP (0.66 mg/dL; reference <0.5 mg/dL), which could not be explained by the brief atrial fibrillation episode lasting approximately 10 min and resolving spontaneously after discontinuation of 5-fluorouracil.

Given the initial ST-segment elevation and severe left ventricular systolic dysfunction, urgent coronary angiography was performed, demonstrating normal coronary arteries and excluding obstructive coronary disease.

Guideline-directed therapy for acute heart failure was initiated, including valsartan 80 mg, eplerenone 25 mg, dapagliflozin 10 mg, transdermal nitro-glycerine 5 mg overnight, and intravenous furosemide 40 mg.

Follow-up ECGs demonstrated sinus rhythm at 70 bpm, abnormal repolarization with QT prolongation and deep T-wave inversion, and complete resolution of ST-segment elevation.

**Figure 2:**
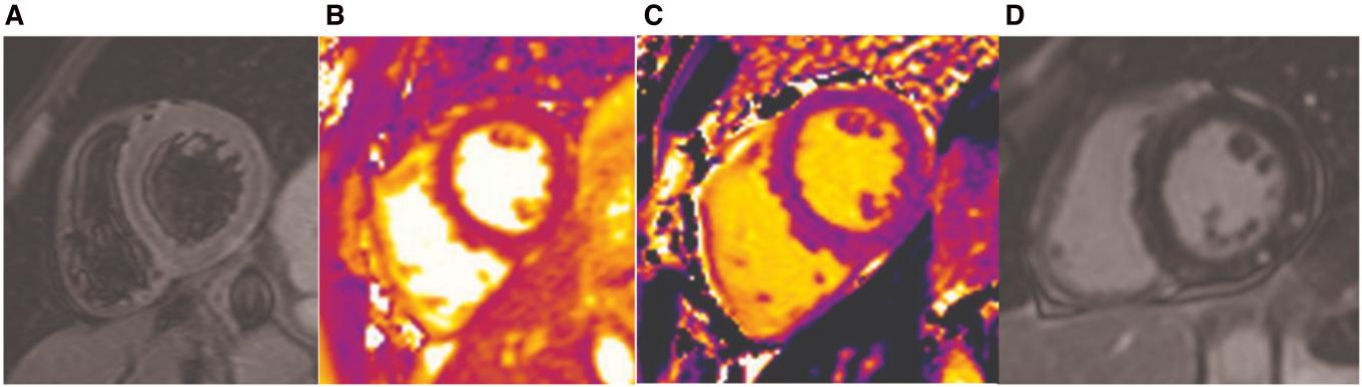
Baseline cardiac magnetic resonance imaging. The combination of diffuse native T1 elevation, borderline T2 prolongation, and non-ischaemic subepicardial late gadolinium enhancement supports the diagnosis of acute inflammatory myocarditis. *(A*) T2-weighted STIR sequence. Mid-ventricular short-axis view demonstrating increased subepicardial signal intensity in the inferolateral wall, consistent with myocardial oedema. *(B*) T2 mapping (T2-prepared bSSFP). Mid-ventricular short-axis view showing globally increased T2 values at the upper limit of normal (51 ± 4 ms), suggesting myocardial oedema (normal reference range for males ≈ 45 ± 2 ms). *(C*) Native T1 mapping (MOLLI sequence). Mid-ventricular short-axis view demonstrating diffusely elevated native T1 values (global T1 1107 ± 56 ms), consistent with diffuse myocardial injury and inflammatory involvement (normal MOLLI T1 reference for males ≈ 983 ± 50 ms). *(D*) Late gadolinium enhancement (PSIR gradient-echo sequence). Mid-ventricular short-axis view demonstrating focal non-ischaemic enhancement with a subepicardial distribution in the basal inferior and inferolateral segments of the left ventricle.

Cardiac magnetic resonance (CMR) imaging (*Figure 2*) showed reduced left ventricular systolic function (LVEF 35%), diffuse myocardial oedema confirmed by elevated T1 and T2 mapping values, and non-ischaemic late gadolinium enhancement with a subepicardial pattern in the basal inferior and inferolateral segments.

No abnormalities were detected on immunological, thrombophilic, or virological testing.

Marked clinical and instrumental improvement was observed within 4 days of therapy initiation. Echocardiography demonstrated normalization of biventricular function (LVEF 60%, LV-GLS −17%, FAC 40%, RV-GLS −19%), with no valvular dysfunction and complete normalization of ECG and laboratory parameters. The patient was discharged 7 days after the index event on optimized medical therapy, including a low-dose beta-blocker (bisoprolol 2.5 mg).

One month later, after multidisciplinary discussion and considering tumour unresectability, young age, and limited therapeutic alternatives, a new cycle of FOLFOX-6 was administered without dose reduction in the coronary intensive care unit under continuous cardiac monitoring.

**Figure 3:**
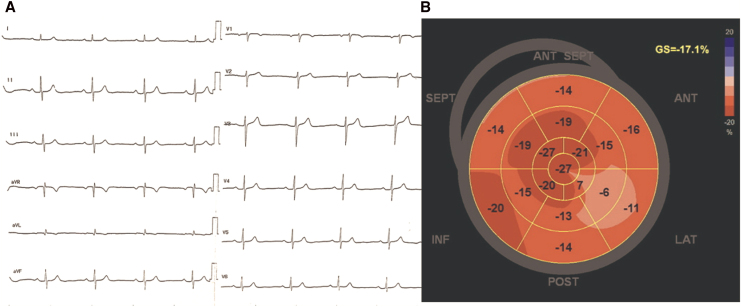
Follow-up electrocardiographic and echocardiographic findings. *(A*) ECG demonstrated normal sinus rhythm with normal axis, intervals, and repolarization, without ST-segment abnormalities or conduction disturbances. *(B*) Transthoracic echocardiography with global longitudinal strain (GLS) assessment: polar map of left ventricular longitudinal strain showing preserved systolic function with a GLS of −17.1%. Segmental strain distribution is homogeneous without focal reduction, indicating normal myocardial deformation. Biventricular dimensions and systolic performance are within normal limits.

During the 48-hour infusion, the patient remained asymptomatic and showed no ECG abnormalities. Serial echocardiography demonstrated stable cardiac function, with a pre-discharge LV-GLS of −17% (*Figure 3A* and *B*). A transient increase in cardiac biomarkers was observed, with hs-troponin I peaking at 124 ng/L and NT-proBNP at 654 ng/L.

The patient was discharged with a recommendation to continue scheduled oncologic therapy under cardiologic surveillance according to high-risk monitoring recommendations.

During subsequent chemotherapy cycles, NT-proBNP and troponin levels remained within normal limits while receiving cardioprotective therapy with an angiotensin receptor blocker, mineralocorticoid receptor antagonist, SGLT2 inhibitor, and beta-blocker (valsartan 80 mg, eplerenone 25 mg, dapagliflozin 10 mg, bisoprolol 2,5 mg).

The patient showed good treatment adherence and tolerated subsequent chemotherapy cycles without clinically relevant adverse effects.

**Figure 4:**
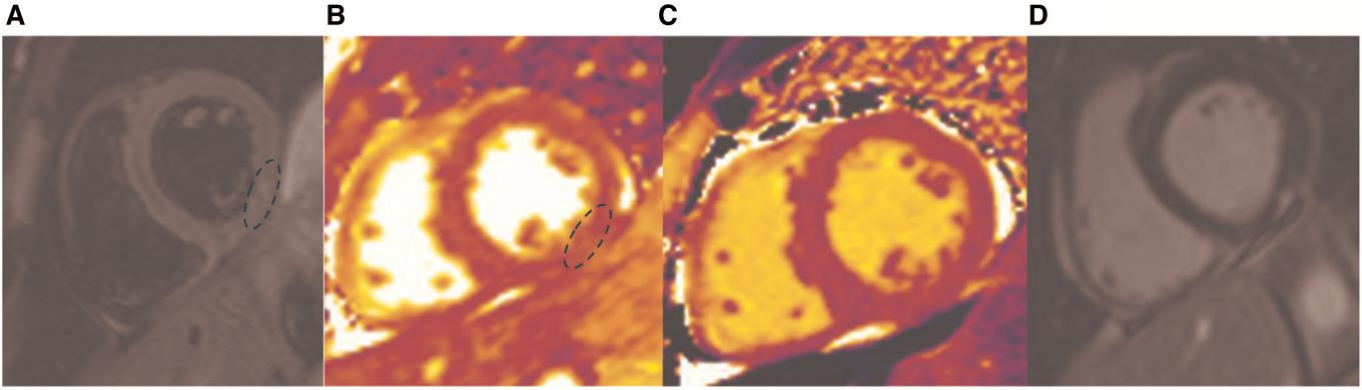
Follow-up cardiac magnetic resonance imaging. Follow-up CMR showed preserved ventricular size and systolic function, substantial reduction of myocardial oedema compared with baseline, and persistent focal non-ischaemic subepicardial late gadolinium enhancement consistent with residual post-inflammatory scar. Region-of-interest analysis was used to further characterize the residual signal alteration on T2-weighted imaging and T2 mapping. *(A*) T2-weighted STIR sequence. Mid-ventricular short-axis view showing markedly reduced myocardial hyperintensity compared with baseline, with mild residual patchy signal alteration in the inferolateral wall (ROI). *(B*) T2 mapping (T2-prepared bSSFP). Mid-ventricular short-axis view showing normal global T2 values (43 ± 5 ms), with a mildly increased focal T2 value at the ROI in the inferolateral wall (50 ± 3 ms), consistent with a possible mild residual regional abnormality. *(C*) Native T1 mapping (MOLLI sequence). Mid-ventricular short-axis view demonstrating normal global native T1 values, indicating resolution of diffuse myocardial injury. *(D*) Late gadolinium enhancement (PSIR gradient-echo sequence). Mid-ventricular short-axis view demonstrating persistent focal non-ischaemic enhancement with subepicardial distribution in the basal inferior and inferolateral segments of the left ventricle, compatible with residual post-inflammatory myocardial scar.

Three months later, after six cycles of FOLFOX-6, follow-up CMR showed non-dilated ventricular chambers with preserved systolic function, substantial reduction of myocardial oedema, normal global T2 mapping values with only a mildly increased focal T2 value at the inferolateral ROI, mild residual patchy signal alteration persisted on T2-weighted imaging, and persistent non-ischaemic LGE in the basal inferior and inferolateral segments (*Figure 4*).

## Discussion

Colon cancer is the third most common malignancy worldwide, accounting for approximately 10% of all cancer diagnoses. Chemotherapy plays a fundamental role in its management, and fluoropyrimidines [5-fluorouracil (5-FU) and oral capecitabine] represent one of the most widely used drug classes across treatment settings.^1^

In this case, a FOLFOX-6 regimen with neoadjuvant intent was administered, consisting of 5-fluorouracil, oxaliplatin, and folinic acid.

Fluoropyrimidines are the second most common class of chemotherapeutic agents associated with cardiotoxicity.^2^ Clinical manifestations frequently include chest discomfort, palpitations, and angina pectoris, the latter being the most reported cardiac adverse event. The proposed underlying mechanism is coronary vasospasm, which is well documented in animal models and reported in clinical series. Less commonly, atrial fibrillation, arrhythmias, myocarditis, pericarditis, heart failure, cardiogenic shock, and cardiac arrest have also been associated with fluoropyrimidine administration.^3^ Importantly, cardiotoxicity may occur even in patients without known cardiovascular disease, and the relationship between baseline cardiovascular risk factors and drug-induced cardiotoxicity remains incompletely understood.^4^ The risk appears to be schedule-dependent, as continuous 5-FU infusion is associated with higher cardiotoxicity rates than bolus administration.^5^

Several mechanisms have been proposed to explain fluoropyrimidine-related cardiotoxicity, including direct cardiomyocyte injury mediated by increased reactive oxygen species production and reduced antioxidant enzyme activity. Metabolites of 5-FU, such as fluoroacetate and fluorocitrate, may increase oxidative stress and impair aerobic metabolism in cardiomyocytes, promoting inflammation and apoptosis.^6^ These processes can lead to coronary vasospasm, endothelial dysfunction, thrombosis, autoimmune phenomena, or direct myocardial injury. Cardiomyocytes may be particularly susceptible to oxidative damage because of their high mitochondrial density and metabolic demand.^7^

Although less frequently reported, cisplatin has also been associated with cardiotoxic effects, including myocarditis, pericarditis, angina, arrhythmias, atrial fibrillation, and myocardial infarction.^8^ Symptomatic cardiotoxicity appears more likely when cisplatin is administered concomitantly with 5-FU, and the risk may increase with cumulative exposure, suggesting a dose-dependent mechanism.^2^

In the present case, during the first cycle of FOLFOX-6 and in the final hours of continuous 5-fluorouracil infusion, the patient developed abdominal pain, nausea, and palpitations associated with transient ST-segment elevation on ECG, severe biventricular dysfunction on echocardiography, and marked elevation of hs-troponin and NT-proBNP. Multimodality imaging enabled the diagnosis of acute toxic myocarditis.

According to the European Society of Cardiology cardio-oncology guidelines, myocarditis is classified as a rare cancer therapy-related cardiovascular toxicity.^2^ These guidelines recommend baseline cardiovascular risk assessment before initiating fluoropyrimidine therapy in all patients, including blood pressure measurement, ECG, lipid profile, HbA1c, and SCORE2/SCORE2-OP risk stratification. A baseline transthoracic echocardiogram is advised in patients with prior cardiovascular disease. Our patient had a low baseline cardiovascular risk profile.

Management of this presentation did not differ from standard treatment of acute myocarditis with severe ventricular dysfunction. Immediate discontinuation of 5-FU was essential, followed by prompt initiation of guideline-directed heart failure therapy. Uridine triacetate, an oral prodrug of uridine approved by the FDA for severe cardiac, haematologic, or neurologic toxicity, represents a specific antidote for severe fluoropyrimidine toxicity. It competitively inhibits fluorouracil-induced cellular injury and is indicated for early-onset severe toxicity or overdose within 96 h of drug administration. Beyond this time window, efficacy decreases substantially. In two open-label studies including 142 patients with severe toxicity or overdose, treatment with uridine triacetate (10 g every 6 h for 20 doses) resulted in the reversal of toxicity in 96% of cases.^9^ In our patient, uridine triacetate was made available as a precautionary measure for subsequent chemotherapy cycles.

Rechallenge with 5-FU after cardiotoxicity is generally discouraged because recurrence may lead to myocardial infarction, cardiogenic shock, or death.^10^ However, in selected patients with suspected vasospasm, rechallenge may be considered with prophylactic nitrates and calcium-channel blockers. Dose reduction and careful cardiac monitoring have also been proposed, and bolus administration appears safer than continuous infusion.^5^

In our patient, continuation of the FOLFOX-6 regimen represented the most effective oncologic strategy to maximize survival and potential operability. A multidisciplinary decision involving cardiologists and oncologists allowed safe continuation of chemotherapy with intensive monitoring and preventive cardioprotective measures.

Strengths of this case include comprehensive multimodality imaging and detailed longitudinal follow-up. Limitations include the single-patient design and the inability to establish causality or generalize findings.

This case underscores the importance of comprehensive cardiovascular assessment for accurate diagnosis of fluoropyrimidine-related cardiotoxicity and highlights the central role of multidisciplinary cardio-oncology teams in both acute management and long-term care, suggesting that carefully selected patients may safely undergo chemotherapy rechallenge under structured monitoring.

## Lead author biography


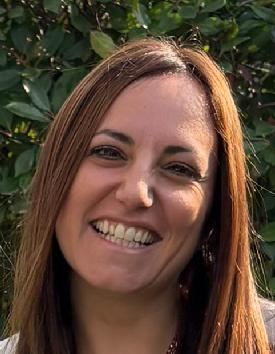
Dr Alessandra Greco earned her medical degree and completed her specialization in cardiovascular disease at the University of Pavia (Italy). She currently serves as a clinical cardiologist at IRCCS Policlinico San Matteo, Pavia, Italy. She has developed significant experience in the Cardioncology and Heart Failure and actively contributes to scientific research.

## Data Availability

The data underlying this article are available in the article.
